# Women’s determinant factors for preferred place of delivery in Dodoma region Tanzania: a cross sectional study

**DOI:** 10.1186/s12978-017-0373-7

**Published:** 2017-09-06

**Authors:** Agatha Fabian Ngowi, Switbert R. Kamazima, Steven Kibusi, Ainory Gesase, Theodora Bali

**Affiliations:** 1 Department of Public Health, College of Health Sciences, Dodoma University, P.O.Box 295, Dodoma, Tanzania; 2 Department of Behavioral Sciences, Muhimbili University of Health and Allied Sciences, P.O Box 65001, Dar-es-salaam, Tanzania; 3 Department of Clinical Nursing, College of Health Sciences, Dodoma University, P.O.Box 295, Dodoma, Tanzania; 4 Department of Anatomy,College of Health Sciences, Dodoma University, P.O BOX 295, Dodoma, Tanzania; 5 Department of Education, Faculty of Humanities and Education, St John University, P.O Box 47, Dodoma, Tanzania

**Keywords:** Maternal health, Delivery place, Determinant factors, Women, Dodoma, Tanzania

## Abstract

**Background:**

Skilled birth attendance is one of the key factors in improving maternal health but less than 50% of women in sub-Saharan African countries do not have the opportunity to be attended to by skilled personnel during childbirth. The aim of the study was to assess the factors determining women’s preference for a place to give birth in Dodoma Region, Tanzania.

**Methods:**

This study employed a cross-sectional survey design using quantitative data collection and analysis methods. Data were collected using structured questionnaire administered to 800 women obtained through multistage random sampling. Multivariable logistic regression model was applied to determine the predictors of place of delivery.

**Results:**

More than three quarters 629(78.6%) respondents had their last delivery in the health facilities while 171(21.4%) had their last delivery at home/on the way to hospital. Reasons for delivering at home include: abrupt occurrence of labour pain, long distance to the health facilities, lack of money to pay for transport and unfriendly experience with the health care providers. Simple logistic regression model indicate that mothers’ education level, number of children, cost of transport the estimated distance to the nearby health facility and occupation were strong predictors of the preferred place of delivery. However, after controlling the potential confounder, the multivariable logistic regression model demonstrated a significant association between delivery at the health facility and the number of children and transport cost**.**

**Conclusion:**

Our findings suggest a need for health care providers to enhance health education to women and their spouses about birth preparedness and the importance of delivering at the health facility. There is also a need for the government to increase the number of health facilities including maternity waiting homes and well trained health workers in both rural and urban areas.

## Plain English summary

This study was conducted to identify the reasons why some women in Dodoma Region choose to give birth at the health facilities and others at home**.**Data were collected from 800 women through face to face interview using structured questionnaire. The reasons why women choose to give birth at home included, sudden occurrence of labour pain, long distance to the health facilities, lack of money to pay for transport and unfriendly experience with the health care providers. Mothers’ education level, number of children, cost of transport, occupation and estimated distance to nearby health facilitywere also among the reasons why women choose to give birth at the health facilities or otherwise. When developing strategies that will influence women’s choice for a safe place to give birth at in Dodoma region, the government and health care providers need to address the issues identified in this study.

## Background

Maternal mortality and morbidity is a global public health concern. Every minute a woman dies from child birth-related complications worldwide [1, 2]. It is estimated that, more than 302,950 women die every year from pregnancy-related causes globally, of which 99% of deaths occur in low income countries. Of those, 50% occur in sub-Saharan Africa [3, 4]. The maternal mortality ratio (MMR) in Tanzania is 410 deaths per 100,000 livebirths [5, 6].

In low income countries, major causes of maternal mortality are preventable if a skilled attendant is present during childbirth. It has been reported that the rates of deliveries attended by skilled health workers are low in developing countries. A study conducted in Bangladesh showed that only 48.9% of women delivered at health facilities and assisted by a skilled attendant [7]. According to Tanzania Ministry of Health and Social Welfare (MOHSW) report [8] the national target for health facility delivery to be attended by skilled personnel was 80%by 2015. However, only 63% of women delivered at the health facilities and assisted by health care providers and 37% delivered at home [9].

One of the strategies for reducing maternal morbidity and mortality in Tanzania is by improving access and timely utilization of maternal health care services [8]. Various efforts have been made by the government of Tanzania to improve utilization of maternal health services for reducing maternal death and disabilities. The government has improved its network of primary health facilities with maternity health care services; conducted health promotion campaigns targeting women of reproductive age and exempted fees for maternal services including delivery at any a government facility [10]. Despite the government’s efforts, the rates of delivery at the health facilities in Tanzania remain low. In Dodoma region, for example, 69% and 31% of deliveries occurred at the health facilities and at home respectively [9]. To enhance the rate of deliveries at the health facilities in the country, there is a need to identify barriers/determinant factors for utilization of maternal health services during delivery among women across all geographical regions. Limited research has been conducted in Dodoma Region to assess factors determining women’s preferred place of delivery, the gap which this study intended to bridge. The objective of this study was to determine factors influencing preferred place of delivery among women in Dodoma Region.

## Methods

### Study area and period

The study was conducted from November 2013 to June 2014 in Dodoma Region, Tanzania.Administratively; Dodoma Region is divided into seven districts namely Kondoa, Bahi, Mpwapwa, Kongwa, Chamwino, Chemba and Dodoma Urban. The study was conducted in Dodoma Urban, Kondoa, Kongwa and Chamwino districts.

According to the 2012 Nation Census [11], the population of Dodoma Region was 2,083,588. The Average Annual Intercensual Growth Rate is 2.1% and population density is 50 people per sq.km. The percentage of women of reproductive age is 44.1% of the total female population in Dodoma Region. The Region has a total of seven hospitals (5 public; 2 private), 32Health Centres (26 public; 6 private) and 269 dispensaries.

### Design and participants

The study employed a cross sectional survey design, targeting women of reproductive age (15–49 years old) who had at least one child in the past five years. Women who were sick or had mental health problems and those who were not willing to participate were excluded from the study.

### Sample size determination and procedure

The sample of 800 women was determined using aformula (N = z^2^ p (1-p)/e^2^) where N = Sample size; Z = Z score for 95% confidence level, which is 1.96; p = Prevalence in previous studies (percentage of women delivered at the health facility in Dodoma was 45.1%); e = tolerable error (set at 5%). The estimated sample size was *N* = 380, 10% was added due to non-response fear and multiplied by a design effect of 2 due to the multistage nature of the sampling method. Therefore, the minimum sample size based on the formula was 798, but a total of 800 participants were studied.

A multistage sampling technique was used to select sample units from the targeted population. In the initial stage, the list of all districts in Dodoma Region was obtained. Dodoma Municipality was selected purposively because it is an urban district with people of different backgrounds. Three other districts were selected randomly namely, Kondoa, Chamwino and Kongwa. In the second stage, two sub districts called (wards) were randomly selected from each of the four selected districts using a table of random numbers and a total of eight wards were obtained. In the third stage, two villages/streets were selected randomly from each of the eight wards using a table of random numbers, making a total of 16 villages/streets. From each of the 16 selected village/streets, all households with women who had children below five years of age were eligible for the study. Therefore, from each of 16 selected villages/streets, 50 participants were selected from the randomly selected households. Within the households, only one respondent was selected. In the households where there were more than one woman qualifying to participate in the study, a woman who had the youngest child was selected assuming she could remember most of the events that occurred during child delivery.

### Data collection technique and data quality control methods

Structured questionnaire with open- and closed-ended questions was prepared in English and translated to Kiswahili. This was administered by two research assistants who collected data via face to face interview techniques. Both research assistants were graduates working as primary school teachers, and underwent training for two days before data collection. Data collection tools were pre-tested to ensure consistency of the variables included in the study. Data recorded was cleaned, edited and coded before data analysis. However, data exploration was done to make sure of its suitability for analysis.

### Statistical analysis

Data were directly entered into Statistical Package for the Social Sciences (SPSS) version 21 software and Statistical analysis was performed using Statistical Analysis System (SAS) version 9.4. All probabilities were two-tailed and *P* values <0.05 > were regarded as significant. To permit quantitative analysis, data were converted into numeric codes representing measurement variables. To answer the research questions, data were analysed based on relationship among the study variables (independent and dependent). Descriptive statistics such as frequency, mean and standard deviation were used to analyse socio-demographic characteristics of participants and preferred place of delivery. Logistic regression analysis was used to determine factors influencing the preferred place of delivery among women in Dodoma Region. Simple logistic regression model was first used to examine the association between each of the background characteristics and the preferred place of delivery. Variables that were statistically significant and biologically plausible in univariate analysis were entered into a multivariable logistic regression model in order to identify the factors associated with the preferred place of delivery, while accounting for other potential confounders. The results of the model were presented using odds ratios (OR) and 95% confidence interval.

## Results

### Characteristic of the study participants

A total of 800 women with children less than five years old were recruited and participated in the study from the four selected districts. The majority of respondents 499 (62.4%) were Gogo by tribe, 193 (24.1%) were Rangi and 108(13.5%) were other tribes. Age-wise, 531 (67.3%) respondents were between 20 and 34 years old; 209 (26.5%) were between 35 and 49 years; 49 (6.1%) were Teenagers between 15 and 19 years.

In terms of marital status, majority of respondents 438 (54.9%) were married, 243 (30.5%) cohabited, 69 (8.6%) were single and 48 (6.0%) were separated/widowed/divorced.Regarding number of children, 171 (21.4%) respondents had 1–2 children, 439 (54.8%) had 3–4 and 190 (23.8%) had five or more children. Results show that 148 (18.5%) women in Dodoma Region had no formal education. A high proportion of women, 562 (70.3%) had primary education while a smaller proportion, about 89(11.1%) had secondary and tertiary education. About 633 (79. 1%) respondents were peasants and 167(20.9%) had formal employment/self employment (Table 1 *).*

**Table 1:** Socio demographic characteristics of respondents in Dodoma Region

Variable	Frequency	Percent
Age in years		
15–19	49	6.2
20–34	531	67.3
35–49	209	26.5
Total	789	100
Ethnic group		
Gogo	499	62.4
Rangi	193	24.1
Others	108	13.5
Total	800	100
Religious belief		
Christian	583	72.9
Muslims	217	27.1
Total	800	100
Marital status		
Married	438	54.9
cohabited	243	30.5
Single	69	8.6
Divorced, widowed separated	48	6.0
Total	798	100
Number of children		
1–2	171	21.4
3–4	439	54.8
5 +	190	23.8
Total	800	100
Education level		
No formal education	148	18.5
Primary education	562	70.3
Secondary /higher education	89	11.1
Total	799	100
Employment status		
Employed	167	20.9
peasant	633	79.1
Total	800	100

### Place of delivery

More than three quarters of the respondents 629 (78.6%) reported to have delivered at the health facilities (dispensaries, health centres and hospitals), while 171 (21.4%) delivered at home/on the way to health facility. Reasons for delivering at home include: abrupt occurrence of labour pain 89(52%); long distance to the health facilities 43 (25.1%); transport unavailable 23 (13.5%); lack of money to pay for transport 9 (5.3%) and unfriendly experience with the health care providers 7(4.1%). Results also showed that majority of women 617(77.1%) were assisted by skilled health care providers during delivery, 135(16.9) by traditional birth attendants, 26(3.2) by relatives and 22(2.8) had no assistance. Some women 12(1.5%) delivered at the health facilities but ended-up with a TBA’s assistance. Majority of respondents 502 (62.8%) reported that both husband and wife decided on the place of delivery, 195(24.4%) reported making the decision on their own, 65(8.1%) reported husbands making the decision and 38(4.8%) reported the relatives made the decision. Regarding estimated distance to the nearby health facilities, 696 (87%) live within five kilometres while 104 (13%) live more than 5 km (Table 2).

**Table 2:** Women’s last delivery place, Assistance during delivery, reasons for home delivery and decision making on delivery place in Dodoma region Tanzania

Variable	Frequency	Percentage
Last delivery take place (*N* = 800)		
Health facility	629	78.6
Home/on the way to hospital	171	21.4
Assistance during delivery (*N* = 800)		
Skilled health care provider	617	77.1
Traditional Birth Attendant	135	16.9
Relatives	26	3.2
Self	22	2.8
Reasons for home delivery (*N* = 171)		
Abruptoccurrence of labour pain	89	52.0
Long distance to the health facility	43	25.1
Transportation unavailable	23	13.5
Lack of money to pay	9	5.3
Unfriendly experience of health care providers	7	4.1
Who decide on delivery place (*N* = 800)		
Both (husband and wife)	502	62.8
Just me	195	24.4
My husband	65	8.1
Relatives	38	4.8
Estimated distance to the nearby health facility		
Within 5 km	696	87
Above 5 km	104	13

### Simple logistic regression model of predictors of the preferred place of delivery

As stated in the methodology section, univariate analysis based on simple logistic regression model was first used to identify the predictors of the preferred place of delivery. The results of the model presented in Table 3 indicated that number of children (*p* < 0.000; mother’s education level (*p* = 0.0126), estimated distance to the nearby health facility (*p* < 0.0001), occupation (*p* = 0.0022) and cost of transport (*p* < 0.0001), were strong predictors of the preferred place to delivery. The results have shown that the preference to deliver at the health facility is inversely related with the number of children a woman has. For women with 1–2 children, the odds of choosing health facility was almost 3 times that of women with at least 5 children (OR = 3.043, *p* < 0.0001). Compared to women with secondary education or higher, those with primary were reported to be significantly less likely to prefer health facility as place to deliver (OR = 0.359, *p* = 0.0051). However, the odds of the preference to deliver at the health facility among women with 3–4 children was about 2 times significantly greater than that of women with 5 or more children (OR = 1.908, *p* = 0.0010). With respect to distance to the nearby health facility, the results revealed that women stayed with 5 km (OR = 2.977, *p* < 0.0001) were significantly more prevalent to prefer to deliver at the health facility in comparison to women who stayed at distance of above 5 km. Occupation of the mother was another predictor of the preferred place of delivery, with employed women havinga significantly higher chance of preferring the health facility as the placed to deliver than peasant women (OR = 2.159, 0.0022).Regarding the cost of transport to the health facility, the results have shown that the preference to deliver at the health facility was significantly lower among women who paid for transport compared to those who did not pay for transport (OR = 0.239, <0.0001).

**Table 3:** Unadjusted Odds ratios (OR) for the predictors of preferredplace of delivery

Effect	OR	95% CI	*P*-Value
Age (Years)			0.0714
15–19	2.497	1.006, 6.194	0.0484
20–34	1.381	0.948, 2.009	0.0922
35–49	Reference		
Mother Education Level			0.0126
No formal education	0.482	[0.216, 1.076]	0.0748
Primary education	0.359	[0.176, 0.735]	0.0051
Secondary or higher	Reference		
Marital Status			0.7400
Married	Reference		
Cohabited	0.840	[0.575, 1.227]	0.3661
Widowed/separated/divorced	0.754	[0.377, 1.510]	0.4257
Single	0.988	[0.525,1.858]	0.9695
Estimated distance to the nearby health facility			
Within 5 km	2.977	[1.926, 4.604]	<0.0001
Above 5 km	Reference		
Number of children			<0.0001
1–2	3.043	[1.783, 5.193]	<0.0001
3–4	1.908	[1.299, 2.802]	0.0010
5+	Reference		
Occupation			
Employed	2.159	[1.319, 3.534]	0.0022
Peasant	Reference		
Incur transport cost			
Yes	0.239	[0.165, 0.345]	<0.0001
No	Reference		
Client Satisfaction			0.4067
Very Good	1.045	[0.473, 2.310]	0.9128
Good	1.144	[0.604, 2.167]	0.6799
Average	1.628	[0.784, 3.381]	0.1916
Not good	Reference		

### Multivariable logistic regression model of predictors of the preferred place of delivery

The results of the multivariable logistic regression model displayed in Table 4 showed that, accounting for possible confounder the effect of mother education (*p* = 0.8813), estimated distance to the nearby health facility(*p* = 0.4666), and occupation (*p* = 0.2921) on the preferred place of delivery were no longer significant. On the other hand, the number of children that a woman has (*p* = 0.0078) and transport cost (*p* < 0.0001) were the significantly potential predictors of the preferred place of delivery. The results of the multivariable logistic regression model indicated that women with 1–2 (aOR = 2.437, *p* = 0.0025) and 3–4 (aOR = 1.554, *p* = 0.0350) children were significantly more likely to prefer health facility as a place to deliver than women with at least 5 children. With regard to cost of transport to the health facility, the results confirmed that the preference for delivering at the health facility was significantly lower among women who paid for transport compared to those who did not pay for transport (OR = 0.270, <0.0001).

**Table 4:** Adjusted Odds ratios (aOR) for the predictors of the preferred place of delivery

Effect	aOR	95% CI	*P*-Value
Mother Education Level			0.8813
No formal education	0.914	[0.375, 2.225]	0.8431
Primary education	0.843	[0.378, 1.881]	0.6769
Secondary or higher	Reference		
Estimated distance to the nearby health facility			0.4666
Within 5 km	0.796	[0.431, 1.470]	
Above 5 km	Reference		
Number of children			0.0078
1–2	2.437	[1.367, 4.346]	0.0025
3–4	1.554	[1.031, 2.342]	0.0350
5+	Reference		
Occupation			0.2921
Employed	1.346	[0.775, 2.337]	
Peasant	Reference		
Incur transport cost			<0.0001
Yes	0.270	[0.157, 0.464]	
No	Reference		

## Discussion

### Place of delivery

The findings from this study indicate that more than three quarters, (78.6%) of respondents had delivered at the health facilities in their last pregnancy. This result is consistent with the findings of studies conducted elsewhere. For instance, a study conducted across the three ecological zones of Ghana showed that75.8% of women from Dodowa, 61.6% from Kintampo, and 89.3% from Navrongo delivered at the health facilities [12]. Another study conducted in the Democratic Republic of Congo showed that high proportion of women (93.8%) delivered at the health facilities [13].

The Kenya Demographic and Health Survey report [14] has also shown that the proportion of women who delivered at the health facilities increased from 43% in 2008–2009 to 61% in 2014. Again, a similar finding was observed from a community-based cross-sectional study conducted in Nepal which showed the changing trends of place of delivery. It was found that within five years’ time interval, the percentage of women who delivered at the health facilities increased from 45.13% to 63.88% [15]. The reasons reported for the increase of institutional delivery were the implementation of various programs along with safe motherhood, free services for institutional delivery, Safe Delivery Incentive Program (SDIP) and establishment of birthing centers in rural areas [12–15].

The 2015–16 TDHS-MIS [9] result showed that the proportion of respondents who delivered at the health facility in the study area is 69%, an increase from 45.1% in TDHS 2010 [16]. The noted increase in health facility delivery in Dodoma Region might be due to the implementation of various reproductive and child health interventions in the community such as improved primary health facilities with maternity health care services and fee exemption for maternal health services [17]. Other contributing factors include on-going health promotion campaigns through media, health care providers and community leaders.

Reasons mentioned by those women who delivered at home such as abrupt labor pain, long distance to the health facilities, unavailability of transport, lack of money to pay and unfriendly experience with health care providers are similar to those given by women in the existing studies [18–20]. The fact that those women who delivered at home are not attended by skilled health professionals may contribute to high maternal morbidity and mortality. However, this study also showed that some women delivered at the health facilities but ended-up with a TBA’s assistance. This may be due to shortage of health care providers at the health facilities especially in the rural areas. All women need access to skilled care during childbirth as timely management and treatment can make the difference between life and death [21]. The government through the Ministry of Health, Community Development, Gender,the Elderly and Children (MoHCDGEC) need to increase the number of well-trained health workers in both rural and urban areas.

### Predictors of the choice of delivery place

The results of the simple logistic regression model indicate that mothers’ education level, number of children, cost of transport, the estimated distance to the nearby health facility and occupation, were strong predictors of the preferred place of delivery. However, after controlling the potential confounder the multivariable logistic regression model demonstrated a significant association between a delivery at the health facility and the number of children and transport cost.

With regard to the education level of a woman, it showed that the likelihood of delivering at the health facility increases with an increase in mother’s education level. Women with secondary education and above were more likely to deliver at health facility compared to those with primary education. This finding is consistent with results of previous studies conducted in other countries [22–25]. The increased likelihood of delivering at the health facility with increase in mother’s education level could be related to increased access to right information concerning maternal health as well as increased women’s autonomy in decision making on the preferred place of delivery. Efforts to improve women literacy should be intensified.

With regard to the number of children, the study results have shown that the likelihood of a woman to deliver at the health facility decreases with an increase in the number of children. This is consistent with the findings of other studies which show that the likelihood of women delivering at the health facility decreased consistently as the number of children ever born increased [26–30]. Perhaps, the likelihood of women with high parity to deliver at the health facility decreased due to increased family responsibility because of large family size which can limit both time and money to seek hospital delivery and other reproductive health care services. Another possible explanation could be due to previous experiences in maternity health services. The attitude of health care providers towards women is a major influence on women’s decision-making about whether or not to use a particular type of maternal health care service. Health care providers need to understand that the manner in which they interact with women, particularly those with high parity, impacts maternal health care utilization, depending on whether a woman perceives her experience as positive or negative. As such, health care providers working in RCH clinics and labour wards need to urgently address care issues in order to improve services provided. Respectful care and education needs to be customized for multiparous women. This care must be free from misconceptions and negative attitudes regarding multi-parity.

The cost of transport and the estimated distance to nearby health facility was inversely related to women’s preferred place of delivery. The finding of this study showed that women who paid transport cost to the health facility and those who stayed long distance to nearby health facilities were less likely to deliver at the health facilities compared to those who did not pay and stayed nearby health facilities. This finding is similar to those from other studies which showed that transport cost and long distances from the household to the health facilities limit women from delivering at the health facilities [31–33]. A study conducted in Kongwa District showed that majority of women either walked or used carts or donkey as a means of transport to the District Hospital due to high transport cost [30]. These challenging conditions reduce the ability of poor people to access maternal health services, which may be contributing to high maternal and child mortality experienced in Tanzania and other developing countries. Though availability of emergency transport and immediate referral services could increase the access to and use of delivery care services by women, often than not there are cost implication as families are compelled to contribute for fuel. The permanent solution is for the government through the MoHCDGEC to increase the number of health facilities including maternity waiting homes in both rural and urban areas.

The results of this study showed that the likelihood of women to deliver at the health facility was higher among women who were employed compared to peasants. This finding is consistent with results of previous study which showed that employed women were more likely to have greater knowledge about pregnancy and childbirth due to freedom of movement and interaction with other people outside the homestead [34, 35]. Employment status can increase women’s economic autonomy and decision making power on preferred place of delivery.

## Conclusion

Despite the increased number of women who delivered at the health facilities in the study area, a notable number of pregnant women still delivered at home, assisted by TBAs or relatives hence increasing the risk of maternal morbidity and mortality. Reasons for home delivery identified in this study were abrupt labor pain, long distance to the health facilities, no means of transportation, lack of money to pay and unfriendly experience with health care providers. Factors significantly associated with preferred place of delivery were mothers’ education level, number of children, transport cost, estimated distance to the health facility and occupation of women. This study adds knowledge to the field of reproductive and child health, especially on the preferred choice of delivery place. These findings also contribute to the knowledge that practitioners, policymakers, planners and other stakeholders need to modify and scale-up existing programmes and interventions and to create policies surrounding maternal health care utilization.

It is therefore recommended that, the health care providers enhance health education to womenand theirspouses about birth preparedness and the importance of delivering at the health facility with assistance of skilled attendant. The government through MoHCDGEC also needs to increase the number of health facilities including maternity waiting homes and well trained health workers in both rural and urban areas. This will prevent women from reverting back to home delivery.

### Study limitation

The study employed cross sectional survey design. The cross-sectional nature of the data does not allow making causal inferences about the relationship between delivery place and the determinant factorsfor preferred place of delivery. However, the study was also conducted in four district of Dodoma Region and findings might not reflect the situation of the rest of the country due to the contextual variations in availability and accessibility of maternal health care services.

## Abbreviations

ANC: Antenatal care
MMR: Maternal Mortality Ratio
MoHCDGEC: Ministry of Health, Community Development, Gender, Elderly and Children
MoHSW: Ministry of Health and Social Welfare
RCH: Reproductive and Child Health
SAS: Statistical Analysis System
SDIP: Safe Delivery Incentive Program
SPSS: Statistical Package for the Social Sciences
TBA: Traditional Birth Attendant
TDHS: Tanzania Demographic and Health Survey
TDHS-MIS: Tanzania Demographic and Health Survey-Malaria Indicator Survey
